# Creating a population-wide Desirable Health Service Utilisation Indicator using linked health and administrative data

**DOI:** 10.23889/ijpds.v9i5.2526

**Published:** 2024-09-18

**Authors:** Harri Doel, Lucy Griffiths, Richard Fry, Rhodri Johnson, Samantha Turner, Ronan Lyons, Jane Lyons

**Affiliations:** 1 Swansea University

## Background and Objective

Healthcare research faces challenges in developing metrics that resonate with the general public or policymakers. We created a Desirable Health Service Utilisation Indicator (DHSUI) to address this gap, centred around New Year’s wishes for survival and non-occurrence of undesired events in the following year, for the population of Wales, UK, following discussions with policy-makers.

## Methods

We accessed and linked individual electronic healthcare records from primary and secondary care sources within the Secure Anonymised Information Linkage (SAIL) Databank. The study used data for all Welsh General Practice registered individuals on the 1st January 2015 with follow-up until residency break, death, or 31st December 2022. The DHSUI was calculated per person per year and quantified the distribution of the population who survive calendar years and do not use selected health services (not admitted to hospital; no emergency department attendance; and not prescribed antibiotics, analgesics, or mental health drugs). We linked area level demographic data to measure socio-economic differences in DHSUI distribution.

## Results

Focussing on 2022, a population of 2,668,977 people were included, with 1,150,347 (43%) identified as meeting the indicator. The proportion of desirable health decreased significantly with age and with increasing socio-economic deprivation. A higher proportion of males (50%) had desirable health compared to females (37%). The most prevalent sub-indicator among those with undesirable health was antibiotic prescriptions accounting for 52% of cases.

## Conclusion

This DHSUI indicator is easy to interpret and will assist policymakers and the public in understanding the magnitude and distribution of desirable healthcare utilisation.

